# The Relationship Between Contraceptive Use and Respiratory Function in Women: A Systematic Review

**DOI:** 10.3390/healthcare13172171

**Published:** 2025-08-30

**Authors:** Aseel Aburub, Mohammad Z. Darabseh, Mozon A. Abzakh, Eman Omar Alhasan, Rahaf Badran, Ala’a Alasmar, Assia BenBraiek, Viktória Prémusz, Márta Hock

**Affiliations:** 1 Department of Physiotherapy, Faculty of Allied Medical Sciences, Applied Science Private University, Amman 11931, Jordan; 2 Department of Physiotherapy, School of Rehabilitation Sciences, University of Jordan, Amman 11942, Jordan; mozonabzakhh@gmail.com; 3 Department of Clinical Nutrition and Dietetics, Faculty of Allied Medical Sciences, Applied Science Private University, Amman 11931, Jordan; i_alhasan@asu.edu.jo; 4 Department of Physiotherapy, Faculty of Applied Medical Sciences, Philadelphia University, Amman 19392, Jordan; rahaf.badran70@gmail.com; 5 Faculty of Dentistry, Applied Science Private University, Amman 11931, Jordan; a_alasmar@asu.edu.jo; 6 Medical and Clinical Laboratory Technology, Faculty of Allied Medical Sciences, Applied Science Private University, Amman 11931, Jordan; a_benbraiek@asu.edu.jo; 7 Institute of Physiotherapy and Sports Science, Faculty of Health Sciences, University of Pécs, H-7621 Pécs, Hungary; premusz.viktoria@pte.hu (V.P.); hock.marta@etk.pte.hu (M.H.); 8 National Laboratory on Human Reproduction, University of Pécs, H-7624 Pécs, Hungary; 9 Physical Activity Research Group, János Szentágothai Research Center, University of Pécs, H-7624 Pécs, Hungary

**Keywords:** oral contraceptive pills, respiratory function, pulmonary function, asthma, birth-control pills

## Abstract

**Background/Objectives:** Hormonal contraceptives are widely used, but their effects on respiratory health remain underexplored. This systematic review examined the impact of oral contraceptive pills (OCPs) on pulmonary function, with an emphasis on asthma-related outcomes and underlying mechanisms. **Methods:** MEDLINE (PubMed), EMBASE (Ovid), CINAHL, AMED, SPORTDiscus, and PEDro were searched from January 2000 to December 2024. Pulmonary outcomes assessed included PEFR, FEV_1_, airway hyperresponsiveness, and exhaled nitric oxide levels. **Results:** Twelve peer-reviewed studies were included. Most studies reported that OCPs do not impair lung function and may even improve respiratory parameters. Women using OCPs showed enhanced peak expiratory flow and reduced symptom variability, particularly in asthma and cystic fibrosis. Potential mechanisms include the stabilization of airway reactivity and modulation of inflammatory pathways. Heterogeneity across study populations and contraceptive types limited a meta-analysis, and few studies stratified outcomes by hormonal composition or comorbidities. **Conclusions:** Hormonal contraceptives may provide protective or regulatory effects on pulmonary function in specific populations. Larger precision-based studies are needed to clarify mechanisms and guide contraceptive counseling for women with respiratory conditions.

## 1. Introduction

Hormonal contraceptives are frequently used by women of reproductive age for menstrual regulation, birth control, and hormonal balance [1]. While their systemic effects on reproductive and metabolic health are well-established, their potential impact on respiratory function remains less clear [1,2]. Estrogens and progestins are key ingredients in combination oral contraceptives, are known to influence a variety of physiological systems, including the immune and respiratory systems, through the modulation of inflammation, smooth muscle reactivity, and mucosal immunity [3].

The respiratory system is vulnerable to changes in hormones throughout the menstrual cycle, especially in diseases like asthma or cystic fibrosis [4,5]. Endogenous sex hormones may change airway inflammation, bronchial responsiveness, and pulmonary function measures, including peak expiratory flow rate (PEFR) and forced expiratory volume in one second (FEV_1_), according to observational and experimental research [6,7,8]. Consequently, it is conceivable that exogenous hormonal regulation via the use of contraceptives might have a positive or negative impact on respiratory outcomes.

Clarifying the pulmonary effects of hormonal contraceptives is important from a clinical and public health standpoint, given their extensive and prolonged usage worldwide [9]. For women who suffer from asthma, chronic obstructive pulmonary disease (COPD), or other long-term respiratory disorders, where respiratory stability is essential to managing their illness and improving their quality of life, this is particularly crucial.

The aim of this systematic review is to evaluate and integrate the existing literature on the relationship between hormonal contraceptive use and respiratory function in women of reproductive age, with a focus on spirometry outcomes. By evaluating the quality of evidence across different study designs, this review aims to provide a clear comprehension of how contraceptive use may affect respiratory health.

## 2. Materials and Methods

This systematic review was prospectively registered on PROSPERO (CRD420251047800) and conducted in accordance with the PRISMA 2020 guidelines. The objective of the review was to evaluate the relationship between contraceptive use and respiratory function in women of reproductive age. Table 1 represents the PRISMA checklist for this study. 

### 2.1. Eligibility Criteria

Studies were considered eligible for inclusion if they investigated females aged 15 to 49 years old and reported associations between any form of hormonal contraceptive use and respiratory outcomes, including spirometric measures such as FEV_1_, FVC, PEFR, respiratory symptoms, airway reactivity, or respiratory infections. The review included observational (cohort, case–control, and cross-sectional) and interventional studies (randomized controlled trials, randomized crossover trials, and pre–post intervention studies). Only studies published in peer-reviewed journals and in English were included. Studies were excluded if they were case reports, conference abstracts, editorials, theses, or letters; if they were animal or in vitro studies; if they did not report any respiratory-related outcomes; or if they focused exclusively on male participants.

Although the search span was broad (1970–2025), we screened older studies carefully for relevance, recognizing that contraceptive formulations have evolved. Where older formulations were included, we noted their differences from modern preparations in the Results and Discussion Sections.

### 2.2. Search Strategy

A comprehensive search strategy was developed and applied to the following databases: MEDLINE (PubMed), EMBASE (Ovid), CINAHL, AMED, SPORTDiscus, and PEDro. The search included studies published from 1 January 1970 to 1 May 2025. The full search strategy is available on PROSPERO. Additional studies were identified by screening reference lists of relevant articles and searching trial and study registers.

To ensure comprehensiveness and reproducibility, Medical Subject Headings (MeSH) and other controlled vocabulary (e.g., EMTREE in Embase) were used. The search strategy involved two main conceptual blocks: S1 (contraception-related terms): “contraception” OR “contraceptives” OR “hormonal contraception” OR “oral contraceptives” OR “combined oral contraceptives” OR “progestin-only pills” OR “birth control” OR “intrauterine device” OR “IUD” OR “injectable contraceptives” OR “hormonal implant” OR “vaginal ring” OR “patch” OR “long-acting reversible contraception” OR “LARC” AND S2 (respiratory terms): “respiratory function” OR “lung function” OR “pulmonary function” OR “FEV_1_” OR “forced expiratory volume” OR “FVC” OR “forced vital capacity” OR “FEV_1_/FVC” OR “peak expiratory flow” OR “spirometry” OR “airway obstruction” OR “asthma” OR “respiratory symptoms”.

Two reviewers (A.A. and M.Z.D.) independently screened the titles and abstracts of all retrieved articles using pre-defined eligibility criteria. Full-text versions of potentially relevant studies were then assessed for inclusion. Discrepancies between reviewers were resolved through discussion, with the involvement of a third reviewer when necessary. Duplicates were identified and removed using EndNote (V21.2) reference management software. To gain a more comprehensive understanding of current research, the grey literature was surveyed via the World Health Organization (WHO) International Clinical Trials Registry platform.

### 2.3. Data Extraction

Data extraction was performed independently by the two reviewers using a standardized form. Extracted information included the author, year of publication, country of origin, study design, sample size, participant characteristics, type of contraceptive used, primary respiratory outcomes assessed, and key findings. Where necessary, the study authors were contacted for clarification or to provide missing data.

### 2.4. Assessment of Risk of Bias

Given the methodological diversity of the included studies, multiple validated tools were employed to assess risk of bias and methodological quality. The Cochrane Risk of Bias 2 (RoB2) tool was used for randomized controlled trials and randomized crossover studies. The AXIS tool was applied to cross-sectional studies. The Newcastle–Ottawa Scale (NOS) was used to assess risk of bias in cohort and case–control studies. The NIH Quality Assessment Tool for Before–After Studies Without Control Groups was applied to pre–post intervention studies. For pharmacokinetic studies, a descriptive checklist adapted from the CONSORT guidelines was used to appraise reporting quality and risk of bias. Risk of bias assessments were performed independently by two reviewers, with disagreements resolved by discussion or by consulting a third reviewer. Studies were assigned an overall quality rating (low, moderate, or high risk of bias).

## 3. Results

### 3.1. Study Section and Characteristics

**Figure 1:**
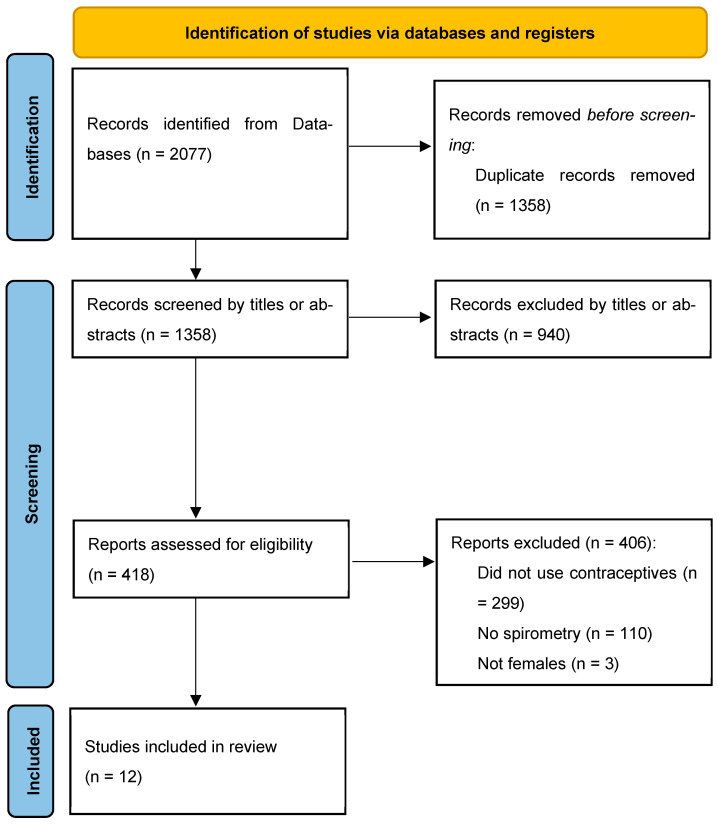
PRISMA chart of the screened records.

A total of 2077 records were identified through database searching. After removing 719 duplicates, 1358 records were screened by title and abstract, of which 940 were excluded for being unrelated, animal studies, or conference abstracts. The full texts of 418 articles were assessed for eligibility, and 406 were excluded for the following reasons: not using contraceptives (n = 299), lacking spirometry data (n = 110), involving non-female participants (n = 3), or being protocols without results (n = 9). Ultimately, twelve studies published between 1973 and 2022 met the inclusion criteria for this systematic review. No relevant trials were found in the grey literature. Figure 1 represents the PRISMA chart of the systematic review. Table 2 represents the data extraction table of the included studies.

The included studies investigated the effects of oral contraceptive pills (OCPs) on pulmonary function across a variety of populations, including healthy women, asthmatics, adolescents with cystic fibrosis, and women with varying hormonal profiles. The sample sizes ranged from small pilot studies of 11–36 participants to large cross-sectional cohorts exceeding 2000 women.

Study designs were diverse, including randomized crossover trials (n = 3), prospective and retrospective cohort studies (n = 3), case–control studies (n = 1), cross-sectional analyses (n = 3), and pre–post intervention studies without control groups (n = 2). The characteristics and outcomes of the included trials are summarized in Table 2, and study quality assessments are presented in Table 3.

Table 2 provides an overview of 12 studies conducted between 1973 and 2022 that explored the relationship between hormonal contraceptive use and pulmonary or immune function in women. The studies vary in design (cross-sectional, cohort, and longitudinal), population (healthy women, asthmatics, and adolescents with cystic fibrosis), and contraceptive formulations (combined oral contraceptives and progestin-only pills). Several studies reported improvements in pulmonary parameters, such as PEFR, FEV_1_, and FEF_25–75_%, among oral contraceptive users, suggesting potential bronchodilatory effects. Others observed reduced bronchial hyperresponsiveness and inflammatory markers, indicating a possible immunomodulatory role. However, some studies found no significant associations, likely due to differences in dosage, duration, hormone composition, or underlying respiratory health. Collectively, the evidence points toward a nuanced relationship where hormonal contraceptives may stabilize respiratory function in certain contexts but not universally across all populations or measures.

Table 3 reviews the methodological quality of twelve studies using validated assessment tools. Four studies demonstrated low risk of bias with strong designs, while seven were rated moderate due to issues like small samples, lack of control groups, or reliance on self-reported data. One study showed moderate-to-high bias. The tools applied included AXIS, NOS, NIH BA, and Cochrane RoB2. Overall, while most studies were transparent and informative, the evidence base has notable limitations. The findings highlight a need for more robust, large-scale, and well-controlled research to better understand the impact of oral contraceptives on pulmonary function.

### 3.2. Pulmonary Function Outcomes

Spirometry measures (FEV_1_, FVC, PEFR, and FEF_25–75_%) were the most commonly reported outcomes across studies. Of the twelve included studies, nine evaluated changes in spirometry parameters with OCP use. Several studies reported improvements in small airway function [16,18], with significant increases in FEF_25–75_% and PEFR among OCP users. Montes et al. (1983) [11] observed an increase in tidal volume at rest and preserved ventilatory performance during exercise. Conversely, three large cross-sectional and cohort studies [12,13,15] found no significant differences in spirometry values between OCP users and non-users after adjusting for potential confounders.

### 3.3. Airway Reactivity and Immune Function

Four studies assessed OCP effects on airway reactivity, and immunologic outcomes [17,20] demonstrated reduced variability in airway reactivity and cough sensitivity among OCP users, particularly during the luteal phase, when endogenous hormonal fluctuations were otherwise expected to increase bronchial reactivity. Vélez-Ortega et al. (2013) [12] reported that OCP users exhibited increased regulatory T cell (iTreg) generation, improved asthma control test (ACT) scores, and decreased exhaled nitric oxide (eNO), suggesting a potential immunomodulatory benefit of OCP use in asthmatics.

### 3.4. Special Populations

In adolescents with cystic fibrosis, Perrissin-Fabert et al. (2020) [19] found that long-term hormonal contraceptive use (>3 years) was associated with significantly higher FEV_1_ compared to non-users, without increasing the risk of exacerbations or antibiotic use. Additionally, Guthikonda et al. (2014) [14] reported that OCP use may modify asthma risk through interactions with genetic polymorphisms (GATA3) and DNA methylation, although further research is needed to clarify these relationships.

### 3.5. Study Quality and Risk of Bias

The quality assessment revealed varying levels of methodological rigor across studies (Table 3). Randomized crossover trials [17,20,22] demonstrated low risk of bias and high internal validity. Large observational cohorts [13,19] were rated low-risk overall but were susceptible to residual confounding inherent in their designs. Pre–post intervention studies [11,18] were rated moderate due to a lack of control groups. Overall, the evidence base was strengthened by consistent findings of improved or stable pulmonary function in OCP users, although heterogeneity in study designs and outcomes precluded a meta-analysis.

## 4. Discussion

This systematic review aimed to synthesize the current evidence from 12 studies regarding the potential impact of contraceptive use on pulmonary function among women of reproductive age. The included studies varied considerably in study design, population characteristics, contraceptive types, and pulmonary outcome measures, contributing to heterogeneity in findings.

The reviewed studies generally indicated that oral contraceptive pills (OCPs) do not impair pulmonary function and, in certain contexts, may provide beneficial effects on specific respiratory parameters. Improvements in airway function were reported in two studies, demonstrated by significant increases in PEFR and FEF_25–75_% among OCP users [16,18]. Similar observations were supported by more recent research indicating improved asthma control and reduced symptom variability in women using hormonal contraceptives [21,23]. Conversely, several large cross-sectional analyses reported no significant association between OCP use and spirometry results [13,15]. In studies examining respiratory variability, OCP use was associated with lower bronchial hyperresponsiveness and cough sensitivity compared to natural hormonal cycles [17,20]. In adolescents with cystic fibrosis, long-term hormonal contraceptive use was linked to significantly higher FEV_1_ compared to non-users [19].

These findings may be explained by several biological mechanisms proposed in the literature. Estrogen and progestin components of OCPs have been shown to modulate airway tone via β_2_-adrenergic pathways, influence mucosal inflammation, and affect pulmonary vascular tone. Estrogen has been associated with smooth muscle relaxation, whereas progestins may reduce mast cell degranulation and histamine release, thereby supporting improved respiratory control [24]. In addition, reduced exhaled nitric oxide and increased regulatory T cell activity among OCP users, as demonstrated by Vélez-Ortega et al. (2013) [12], suggest a potential immunomodulatory and anti-inflammatory role. Such mechanisms provide a plausible explanation for the stability in pulmonary function and reduction in bronchial hyperresponsiveness observed in women using OCPs.

### 4.1. Biological and Pharmacological Mechanisms

Hormonal contraceptives may affect the respiratory system through multiple biological mechanisms. For example, estrogen and progestin modulate airway tone via β2-adrenergic pathways, influence mucosal inflammation, and affect pulmonary vascular tone. Vélez-Ortega et al. (2013) [12] reported reduced exhaled nitric oxide and increased levels of regulatory T cells among OCP users, suggesting an immunomodulatory and anti-inflammatory impact. This is consistent with immunopharmacological models proposed by Borkar et al. (2022) [25], which suggest that sex hormones regulate both innate and adaptive immune responses in asthma.

In addition, estrogen has been linked to smooth muscle relaxation in the airways, while progestins may reduce mast cell degranulation and histamine release, further contributing to improved respiratory control [24]. These biological effects reinforce the clinical observations of stable or improved pulmonary function among OCP users.

Some included studies investigated older, higher-dose contraceptive formulations, which may not reflect the current practice. However, these were retained for historical context, and their differences from modern low-dose formulations were acknowledged when interpreting the findings.

### 4.2. Demographic and Clinical Subgroups

This review highlights that the effects of OCPs may be particularly evident in women with pre-existing respiratory conditions, such as cystic fibrosis. Taylor-Cousar et al. (2023) [26] reported that CF adolescents using hormonal contraception for over three years achieved significantly higher FEV_1_ at age 18 (85.2 % predicted vs. 71.1 %; *p* = 0.043), with no increase in pulmonary exacerbations or hospital admissions. Holtrop et al. (2021) [27] conducted a prospective pilot study on adult women with CF and found that OCP initiation was associated with reductions in sputum inflammatory biomarkers and improvements in lung function, without increased exacerbation frequency [28]. These clinical observations are further supported by population-level registry reviews suggesting overall safety and potential non-contraceptive pulmonary benefits of hormonal contraception in CF, including lower antibiotic usage and fewer pulmonary exacerbations in some cohorts [28].

Additionally, Guthikonda et al. (2014) [14] identified gene–environment interactions involving hormone exposure and asthma risk. This suggests that certain individuals may be genetically predisposed to respond differently to exogenous hormones, underscoring the need for personalized treatment approaches. Women with severe premenstrual asthma (PMA), for instance, may particularly benefit from hormonal regulation.

### 4.3. Strengths and Limitations

The major strengths of this review include its comprehensive search approach, adherence to PRISMA guidelines, and inclusion of diverse study designs. Incorporating observational, cross-sectional, and longitudinal data enriched the depth and scope of the synthesis.

However, several limitations must be acknowledged. Many studies had small sample sizes and no control groups. A meta-analysis was not feasible due to the heterogeneity in contraceptive types, doses, population, and respiratory outcome measures. In addition, the reliance on English-language studies may have led to the exclusion of relevant international research, potentially introducing language and publication bias.

A key limitation of the current body of evidence is the lack of stratification by hormone type (e.g., ethinylestradiol vs. natural estradiol), delivery method (oral, patch, and injection), as it only focused on oral contraceptives, and individual susceptibility (e.g., genetic profiles and baseline lung function). Moreover, many studies failed to control for confounding factors such as smoking, BMI, or environmental exposure. Additionally, potential publication bias and the underrepresentation of non-English studies may limit the comprehensiveness and generalizability of the findings. Another limitation of the current evidence is the heterogeneity of hormonal formulations. Differences in estrogen dose (e.g., ethinylestradiol vs. natural estradiol) and progestin type may lead to variable respiratory outcomes; yet, most studies did not stratify their results by formulation. This variability may partially explain the inconsistent findings across studies.

### 4.4. Future Research Directions

Future studies should prioritize well-powered randomized controlled trials and prospective cohort studies that address these limitations. Special emphasis should be placed on hormonal composition, dosage, duration of use, and patient-specific characteristics. Stratification by respiratory phenotype, genetic profile, lifestyle [29,30], and comorbidity status will also enhance precision in evaluating outcomes.

The integration of respiratory biomarkers (e.g., FeNO and cytokines), hormonal profiling, and lung imaging may offer greater mechanistic clarity [31]. Furthermore, future research should involve diverse ethnic, socioeconomic, and geographic populations to improve generalizability and equity in clinical guidance. Collaborative interdisciplinary efforts across pulmonology, gynecology, immunology, and genomics are essential to develop a comprehensive understanding of how hormonal contraceptives affect respiratory health in both healthy and clinically vulnerable women [32].

## 5. Conclusions

This systematic review highlights a growing body of evidence suggesting that hormonal contraceptives, particularly oral formulations, do not impair pulmonary function and may, in certain subgroups, offer regulatory benefits. These effects appear to be most pronounced among women with pre-existing respiratory conditions such as asthma or cystic fibrosis, where hormonal modulation may attenuate symptom variability and improve airway stability.

The observed respiratory benefits are likely mediated through complex hormonal, immunological, and pharmacological mechanisms, including the stabilization of cyclical hormonal fluctuations, reduction in airway inflammation, and modulation of β_2_-adrenergic pathways. These mechanistic insights support the clinical observation of improved peak expiratory flow and reduced bronchial hyperresponsiveness among OCP users.

However, the current evidence remains limited by methodological heterogeneity, small sample sizes, and insufficient stratification by contraceptive type, hormone dose, delivery method, and patient phenotype. To advance evidence-informed practice, future studies must adopt robust research designs, including randomized controlled trials and longitudinal cohorts with the integration of respiratory biomarkers, hormonal profiling, and genetic susceptibility markers.

Ultimately, personalized contraceptive strategies that account for both reproductive and respiratory health will be essential. Interdisciplinary research spanning pulmonology, gynecology, immunology, and pharmacology will be key to developing tailored and equitable approaches for women with diverse clinical needs. These efforts will ensure that contraceptive counseling not only supports reproductive autonomy but also promotes broader dimensions of women’s health, including respiratory well-being.

## Figures and Tables

**Table 1:** PRISMA checklist for this study. Source: [10]. This work is licensed under CC BY 4.0. To view a copy of this license, visit https://creativecommons.org/licenses/by/4.0/.

Section and Topic	Item #	Checklist Item	Location Where Item Is Reported
**Title**			
Title	1	Identify the report as a systematic review.	1
**Abstract**			
Abstract	2	See the PRISMA 2020 for the Abstract checklist.	1–2
**Introduction**			
Rationale	3	Describe the rationale for the review in the context of the existing knowledge.	2
Objectives	4	Provide an explicit statement of the objective(s) or question(s) the review addresses.	2
**Methods**			
Eligibility criteria	5	Specify the inclusion and exclusion criteria for the review and how studies were grouped for the syntheses.	2–3
Information sources	6	Specify all databases, registers, websites, organizations, reference lists, and other sources searched or consulted to identify studies. Specify the date when each source was last searched or consulted.	3–4
Search strategy	7	Present the full search strategies for all databases, registers, and websites, including any filters and limits used.	3
Selection process	8	Specify the methods used to decide whether a study met the inclusion criteria of the review, including how many reviewers screened each record and each report retrieved, whether they worked independently, and, if applicable, details of automation tools used in the process.	3–4
Data collection process	9	Specify the methods used to collect data from reports, including how many reviewers collected data from each report, whether they worked independently, any processes for obtaining or confirming data from study investigators, and, if applicable, details of automation tools used in the process.	3
Data items	10a	List and define all outcomes for which data were sought. Specify whether all results that were compatible with each outcome domain in each study were sought (e.g., for all measures, time points, and analyses), and if not, the methods used to decide which results to collect.	3
	10b	List and define all other variables for which data were sought (e.g., participant and intervention characteristics and funding sources). Describe any assumptions made about any missing or unclear information.	3
Study risk of bias assessment	11	Specify the methods used to assess risk of bias in the included studies, including details of the tool(s) used, how many reviewers assessed each study and whether they worked independently, and, if applicable, details of automation tools used in the process.	3
Effect measures	12	For each outcome, specify the effect measure(s) (e.g., risk ratio and mean difference) used in the synthesis or presentation of results.	3–4
Synthesis methods	13a	Describe the processes used to decide which studies were eligible for each synthesis (e.g., tabulating the study intervention characteristics and comparing against the planned groups for each synthesis (item #5)).	4
	13b	Describe any methods required to prepare the data for presentation or synthesis, such as handling of missing summary statistics or data conversions.	3–4
	13c	Describe any methods used to tabulate or visually display the results of individual studies and syntheses.	3–4
	13d	Describe any methods used to synthesize results and provide a rationale for the choice(s). If a meta-analysis was performed, describe the model(s), method(s) to identify the presence and extent of statistical heterogeneity, and software package(s) used.	3–4
	13e	Describe any methods used to explore possible causes of heterogeneity among study results (e.g., subgroup analysis and meta-regression).	NA
	13f	Describe any sensitivity analyses conducted to assess the robustness of the synthesized results.	NA
Reporting bias assessment	14	Describe any methods used to assess the risk of bias due to missing results in a synthesis (arising from reporting biases).	3
Certainty assessment	15	Describe any methods used to assess certainty (or confidence) in the body of evidence for an outcome.	3–4
**Results**			
Study selection	16a	Describe the results of the search and selection process, from the number of records identified in the search to the number of studies included in the review, ideally using a flow diagram.	3–4
	16b	Cite studies that might appear to meet the inclusion criteria but were excluded, and explain why they were excluded.	4
Study characteristics	17	Cite each included study and present its characteristics.	4
Risk of bias in studies	18	Present assessments of risk of bias for each included study.	7
Results of individual studies	19	For all outcomes, present the following information for each study: (a) summary statistics for each group (where appropriate) and (b) an effect estimate and its precision (e.g., confidence/credible interval), ideally using structured tables or plots.	5–6
Results of syntheses	20a	For each synthesis, briefly summarize the characteristics and risk of bias among contributing studies.	5–7
	20b	Present the results of all statistical syntheses conducted. If a meta-analysis was conducted, for each study, present the summary estimate and its precision (e.g., confidence/credible interval), as well as measures of statistical heterogeneity. If comparing groups, describe the direction of the effect.	NA
	20c	Present the results of all investigations of possible causes of heterogeneity among the study results.	NA
	20d	Present the results of all sensitivity analyses conducted to assess the robustness of the synthesized results.	NA
Reporting biases	21	Present the assessments of risk of bias due to missing results (arising from reporting biases) for each synthesis assessed.	NA
Certainty of evidence	22	Present the assessments of certainty (or confidence) in the body of evidence for each outcome assessed.	NA
**Discussion**			
Discussion	23a	Provide a general interpretation of the results in the context of other evidence.	9–10
	23b	Discuss any limitations of the evidence included in the review.	10
	23c	Discuss any limitations of the review processes used.	10
	23d	Discuss the implications of the results for practice, policy, and future research.	10
**Other Information**			
Registration and protocol	24a	Provide registration information for the review, including the register name and registration number, or state that the review was not registered.	2
	24b	Indicate where the review protocol can be accessed, or state that a protocol was not prepared.	NA
	24c	Describe and explain any amendments to information provided at registration or in the protocol.	NA
Support	25	Describe sources of financial or non-financial support for the review, as well as the role of the funders or sponsors in the review.	11
Competing interests	26	Declare any competing interests of the review authors.	11
Availability of data, code, and other materials	27	Report which of the following are publicly available and where they can be found: template data collection forms; data extracted from included studies; data used for all analyses; analytic code; and any other materials used in the review.	11

**Table 2:** Summary of trials investigating the effects of oral contraceptive pills on pulmonary function.

Author (Year)	Location	Sample Size	Age (Mean ± SD)	Contraceptive Pills Used	Primary Outcomes	Key Findings	Effect Summary
Montes et al. (1983) [11]	Hawaii, USA	12	Mean 23 (21–30)	1 mg norethindrone + 0.035 mg mestranol; 1 mg ethynodiol diacetate + 0.050 mg ethinyl estradiol	VC, VT, FEV_1.0_, MMEF; VO_2_, VE	↑ VT at rest (*p* = 0.01); ↑ VE with exercise; ventilatory performance preserved	Positive
Vélez-Ortega et al. (2013) [12]	Kentucky, USA	13 (6 OC, 7 non-OC)	OC: 28 (21–34)	Combination OCs (not specified)	FEV_1_s, ACT, eNO, iTregs	OCP users: ↑ iTregs, ↑ ACT, ↓ eNO; no difference in FEV_1_s	Positive
Freedman and Anderson (1973) [13]	California, USA	2066	15–60 (median 39.5)	Mestranol + norethindrone (mostly)	FVC, FEV_1.0_, FEV_0.5_, PEFR	No spirometric differences in OCP use after adjusting for confounders	Neutral
Guthikonda et al. (2014) [14]	UK/USA	245 (subset of 660)	Mean 18	Type/dose not specified	DNA methylation (GATA3), asthma status	OCP use × GATA3 SNPs → ↑ asthma risk (with high methylation)	Negative
Chan et al. (2022) [15]	Australia	1278	Mean 44.6–45.3	36% used OCs or hormone therapy	Spirometry; respiratory questionnaires	Early menarche, PCOS, parity linked to ↓ FEV_1_/FVC; COVID impacted data collection	Negative
Kumar et al. (2011) [16]	India	100 (50 OC, 50 non-OC)	29.84 ± 4.37 (OC)	MALA-N: levonorgestrel + ethinylestradiol	PEFR, FEF_25–75_, FEV_1_%, FVC	OCP users: ↑ PEFR, FEF_25–75_, FEV_1_% (*p* < 0.001); suggests ↓ small airway resistance	Positive
Tan et al. (1997) [17]	Dundee, UK	18 (9 OC, 9 natural cycle)	Mean 24 (±6)	Various monophasic/triphasic OCPs (ethinylestradiol + progestins)	AMP reactivity (PC_20_), PEFR	OCP users: stable PC_20_ across cycle; natural cycles: ↑ reactivity and PEFR variability during luteal phase	Positive
Strinić and Eterović (2003) [18]	Split, Croatia	36 healthy women	Mean 28.7 (24–35)	EE 35 µg + norgestimate 250 µg for 6 months	FVC, FEV_1_, PEFR, FEF_25_/_50_/_75_	All spirometry indices improved (↑ 6.5–15%); FEF_25_ ↑ the most (small airway improvement)	Positive
Perrissin-Fabert et al. (2020) [19]	Montreal, Canada	127 adolescent girls with CF (64 HC users)	13–18 years	Mixed (COCs, progestin-only, ring, injection)	FEV_1_, hospitalization, antibiotic use	FEV_1_ ↑ in HC >3 years vs. non-HC (85% vs. 71%, *p* = 0.043); no ↑ in exacerbations or antibiotic use	Positive
Kavalcikova-Bogdanova et al. (2018) [20]	Slovakia	22 (11 OC, 11 MC)	OC: 22.4 ± 0.4; MC: 23.7 ± 1.3	Monophasic OCs: ethinyl estradiol + dienogest	Cough sensitivity (C2, C5), urge-to-cough, FeNO, FEV_1_/FVC	OCP users showed no significant variation in cough reflex sensitivity; the MC group had ↑ sensitivity in the luteal phase	Neutral
Tan et al. (1998) [21]	Dundee, UK	11 female asthmatics	Mean 25 (19–40)	Various COCs (mono/triphasic: EE + various progestins)	β_2_-adrenoceptor parameters (Bmax, Kd, Emax), FEV_1_, FEF_25–75_%	No effect of OCP on β_2_-adrenoceptor regulation or bronchodilator response	Neutral
Wong et al. (1998) [22]	Illinois, USA	21 healthy women	32 ± 8	30 µg EE + 0.15 mg LNG (Nordette)	Pharmacokinetics (EE and LNG)	ABT-761 ↓ Cmax, and the AUC of EE was significantly affected; mild ↓ in LNG; potential for drug interaction	Negative

VC: Vital Capacity; VT: Tidal Volume; FEV_1_: Forced Expiratory Volume in 1 s; MMEF: Maximal Mid-Expiratory Flow; VO_2_: Oxygen Consumption; VE: Minute Ventilation; ACT: Asthma Control Test; eNO: Exhaled Nitric Oxide; iTregs: Induced Regulatory T cells; FVC: Forced Vital Capacity; PEF/PEFR: Peak Expiratory Flow/Peak Expiratory Flow Rate; PCOS: Polycystic Ovary Syndrome; AMP: Adenosine Monophosphate; PC_20_: Provocative Concentration causing 20% FEV_1_ drop; FEF_25–75_: Forced Expiratory Flow between 25 and 75%; CF: Cystic Fibrosis; HC: Hormonal Contraceptive; MC: Menstrual Cycle (non-OC); FeNO: Fractional Exhaled Nitric Oxide; OCP/COC: Oral Contraceptive Pill/Combined Oral Contraceptive; EE: Ethinylestradiol; LNG: Levonorgestrel; AUC: Area Under Curve (pharmacokinetics); Cmax: Maximum Serum Concentration. ↓: decreased; ↑: increased.

**Table 3:** Quality assessment of included studies.

Author (Year)	Study Design	Assessment Tool Used	Quality Rating/Risk of Bias *	Notes
Montes et al. (1983) [11]	Pre–post (before–after, no control)	NIH BA tool	Moderate	Small sample (n = 12); no control group
Vélez-Ortega et al. (2013) [12]	Cross-sectional	AXIS	Moderate	Small pilot sample; well-reported methods
Freedman and Anderson (1973) [13]	Large cross-sectional	AXIS	Low	Very large sample; adjustments made for confounders
Guthikonda et al. (2014) [14]	Observational cohort	NOS—cohort	Moderate to High	Genetic/methylation study; some risk of confounding
Chan et al. (2022) [15]	Cross-sectional from cohort	AXIS	Moderate	COVID impact on data; self-report limitations
Kumar et al. (2011) [16]	Case–control (OCP vs. non-OC)	NOS—case–control	Moderate	Well-matched groups; small sample size
Tan et al. (1997) [17]	Controlled crossover	Cochrane RoB2	Low	Well-conducted crossover design
Strinić and Eterović (2003) [18]	Before–after	NIH BA tool	Moderate	No control group; modest sample
Perrissin-Fabert et al. (2020) [19]	Retrospective cohort	NOS—cohort	Low	Large sample; good reporting; some retrospective bias possible
Kavalcikova-Bogdanova et al. (2018) [20]	Randomized crossover	Cochrane RoB2	Low	Good randomization and crossover control
Tan et al. (1998) [21]	Controlled observational	AXIS or ROBINS-I	Moderate	Small sample; reasonable control of confounders
Wong et al. (1998) [22]	Randomized crossover PK	Descriptive checklist	Low	Well-reported PK study; no clinical outcomes

AXIS = Appraisal Tool for Cross-Sectional Studies; NOS = Newcastle–Ottawa Scale; NIH BA tool = NIH Quality Assessment Tool for Before–After Studies Without Control Groups; RoB2 = Cochrane Risk of Bias 2; ROBINS-I = Risk of Bias In Non-Randomized Studies of Interventions; PK = Pharmacokinetics. * Key for quality: Low = Low risk of bias and high methodological quality; Moderate = Some limitations but results likely valid; and High = High risk of bias/low quality.

## Data Availability

The data that support the findings of this study are available within the manuscript.
